# Radiation Protection Principles Observance in Mammography Divisions in Shiraz

**DOI:** 10.5812/ircmj.2004

**Published:** 2012-12-06

**Authors:** Zahra Siavashpour, Simin Mehdizadeh, Afrooz Farshadi, Milad Baradaran-Ghahfarokhi

**Affiliations:** 1 Radiation Research Center and Medical Radiation Engineering, Mechanical Engineering Department, Shiraz University, Shiraz, IR Iran; 2 Medical Physics and Medical Engineering Department, School of Medicine, Isfahan University of Medical Sciences, Isfahan, IR Iran

**Keywords:** Mammography, Radiation Protection, Radiography

Dear Editor,

Breast cancer is the second most common neoplasia and the first leading cause of cancer death among women in the world (1). Early diagnosis of breast cancer plays a critical role in reducing mortality rates and improving the patient’s prognosis (2, 3). Mammography is an extremely useful technique for detection of breast cancer (4). An important goal in this imaging modality is to obtain the best diagnostic information by delivering the least radiation (5, 6).

Some protocols by International Atomic Energy Agency (IAEA) are involved in the process of optimizing the radiation used for imaging, which are related to the selection of appropriate imaging equipment, evaluation of equipment performance in the context of quality assurance programs, radiation protection principles and education of medical and technical staff on the appropriate imaging procedures and protocols (7, 8). Considering these protocols, it is essential to have comprehensive knowledge, attitude and practice of mammography in the divisions, emphasize on the importance of its implementation as a routine and preventive measure for early diagnosis of breast cancer (1, 4).

The aim of this study was to investigate the radiation protection principles observance and to evaluate the existing status of radiological practice and equipment performance according to the radiation protection protocols established by IAEA in mammography division in Shiraz. According to the best of our knowledge, there was no evidence for the same study in Iran. A questionnaire-based study was carried out in 5 diagnostic mammography divisions of Shiraz namely Namazi, Faghihi, Hafez, Zeinabieh, and MRI. All of the 30 women staff were interviewed between February 2008 to March 2008. All of them were asked to participate in the study. The refusal rate to participate was very low.

The above mentioned questionnaire consisted 5 main categories. First, the audiences were asked about radiation dose measurement units. The second part was about the Annual Maximum Permissible Dose (AMPD) (5, 6), the necessary training in regards to the procedures and quality assurance and radiation programs at the beginning of the work made the third and fourth ones. The fifth was about renewing the licenses. Then the questionnaires were reviewed for information quality and legitimacy, and corrections were made as needed. After reviewing the questionnaires, statistical analysis was performed using SPSS software (Version 10) by descriptive statistics.

**Figure 1:**
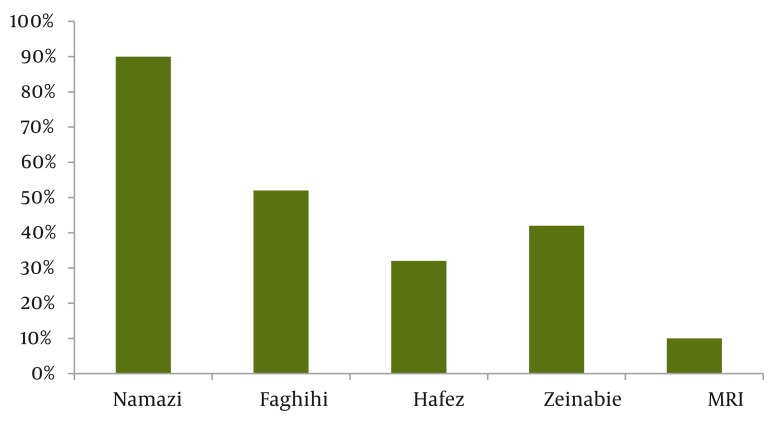
Status of Personnel Training in Quality Assurance and Radiation Programs

We found that in mammography divisions in Shiraz, 47% of the staffs had not passed necessary training in regards to the procedure nor quality assurance and radiation program at the beginning of their work (Figure 1). About 80% of personnel had not their licenses renewed by attending the training classes again. Moreover, 80% of the machinery and equipment were not being regulated nor monitored as often as needed. Most of the radiation workers were aware of radiation dose measurement units and AMPD (> 95%). In addition, we found that in 80% of the divisions, there was no special radiation protection shield used for the patients. This study showed that an adequate training of staffs in mammography divisions was required to reduce the patient's radiation dose. Implementation of radiation protection courses and education of practical issues, including radiation dose received by patients and radiation safety, during medical education programs could be an effective method to reduce the patient's dose in medical exposures.

Our findings are similar to other studies in the literature (4, 9). In our study, the interviewed staffs were from five different divisions in Shiraz. Therefore, our results may not apply throughout Iran, but it seems that most divisions have the same problems (4, 10). The lack of awareness becomes particularly pertinent when we consider the number of staffs who received inappropriate training. Further investigation in other parishes of Iran is suggested. Combined to the data of the present study, both could provide better understanding of the existing status of radiological practice and equipment performance in mammography divisions.
